# The Neurovestibular Challenges of Astronauts and Balance Patients: Some Past Countermeasures and Two Alternative Approaches to Elicitation, Assessment and Mitigation

**DOI:** 10.3389/fnsys.2016.00096

**Published:** 2016-11-22

**Authors:** Ben D. Lawson, Angus H. Rupert, Braden J. McGrath

**Affiliations:** ^1^U.S. Army Aeromedical Research Laboratory (USAARL)Fort Rucker, AL, USA; ^2^Faculty of Education, Science, Technology and Maths, University of CanberraCanberra, ACT, Australia

**Keywords:** vestibular, balance, space adaptation, orientation, falling, tactile, sway, vertigo

## Abstract

Astronauts and vestibular patients face analogous challenges to orientation function due to adaptive exogenous (weightlessness-induced) or endogenous (pathology-induced) alterations in the processing of acceleration stimuli. Given some neurovestibular similarities between these challenges, both affected groups may benefit from shared research approaches and adaptation measurement/improvement strategies. This article reviews various past strategies and introduces two plausible ground-based approaches, the first of which is a method for eliciting and assessing vestibular adaptation-induced imbalance. Second, we review a strategy for mitigating imbalance associated with vestibular pathology and fostering readaptation. In discussing the first strategy (for imbalance assessment), we review a pilot study wherein imbalance was elicited (among healthy subjects) via an adaptive challenge that caused a temporary/reversible disruption. The surrogate vestibular deficit was caused by a brief period of movement-induced adaptation to an altered (rotating) gravitoinertial frame of reference. This elicited adaptation and caused imbalance when head movements were made after reentry into the normal (non-rotating) frame of reference. We also review a strategy for fall mitigation, viz., a prototype tactile sway feedback device for aiding balance/recovery after disruptions caused by vestibular pathology. We introduce the device and review a preliminary exploration of its effectiveness in aiding clinical balance rehabilitation (discussing the implications for healthy astronauts). Both strategies reviewed in this article represent cross-disciplinary research spin-offs: the ground-based vestibular challenge and tactile cueing display were derived from aeromedical research to benefit military aviators suffering from flight simulator-relevant aftereffects or inflight spatial disorientation, respectively. These strategies merit further evaluation using clinical and astronaut populations.

## Introduction

Terrestrial spatial orientation and balance are maintained by overlapping multi-sensory information that is continually updated and cross-referenced. When processing of stimuli associated with body motion is altered, immediate changes in cognitive perceptions of self-orientation and motion occur, along with reflexive vestibulo-spinal, -ocular, and -autonomic responses (Baloh and Halmagyi, 1996; Baloh and Honrubia, 2001; Brandt et al., 2014). Altered processing in these domains of functioning is usually elicited by two main categories of sensorimotor challenge: (A) endogenous maladies that alter sensorimotor relationships (e.g., vestibular injury, disease, or aging); and (B) exogenous alterations of the gravitoinertial force environment, which occur during aircraft flight, acceleration in laboratory/simulator devices, or spaceflight.

Regardless of which challenge causes altered processing of body motion, there is an initial period of disruption of functional abilities, leading to disorientation, imbalance, disruption of gaze control, or motion sickness symptoms. The process of disruption and subsequent adaptation is somewhat similar for patients recovering from many types of vestibular pathology as for healthy people adapting to the vestibular challenge posed by exposure to a flight simulator or a prolonged voyage at sea or into space. Once adaptation is achieved by sensorimotor recalibration to these new processing demands, coordination and well-being is restored. In the case of recovery from endogenous vestibular deficits, such adaptation represent an unqualified positive outcome. Unfortunately, in the case of adaptation to sea or space travel, the initial compensation confers resistance only to the sensorimotor effects of the voyage *per se* (i.e., the motions of the sea or the altered vestibular inputs caused by weightlessness). The sea or space voyager must then go through a second process of adaptation upon returning to Earth after a prolonged voyage. This is because the voyager must readapt to walking on a terrestrial substrate while being subjected to a steady and unidirectional acceleration due to Earth’s gravity. The same sequence of adaptation and readaptation accompanies many other sensory rearrangements, such as prolonged exposure to a military flight simulator (which alters the normal relationship between visual, vestibular and somatosensory inputs).

This review article compares the processes of disruption and adaptation associated with endogenous vestibular pathology to those associated with exogenous changes from the normal gravitational force level occurring during spaceflight. We briefly discuss how a preliminary ground-based gravitoinertial force alteration strategy might prove useful as a proxy for the induction and measurement of balance deficits caused by vestibular maladies or aftereffects of spaceflight. We also describe sensitive tests for balance deficits which have spun off from such work.

In addition to discussing a possible balance deficit induction and assessment strategy, we discuss countermeasures. We review a few of the plausible strategies for mitigating imbalance due to space adaptation, including a sensory cueing strategy (and associated device) that we helped to initiate. The device is a tactile display that is known to reduce in-flight spatial disorientation. Explorations were made of its potential usefulness for avoiding imbalance and falls following vestibular pathology. We conclude that this strategy is worth exploring further, to determine if it will help with the space adaptation syndrome during/after spaceflight. To date, little is known concerning the potential space-related benefits of this strategy, particularly post-flight (van Erp and van Veen, 2006). We turn to the first of our main topics below: the comparison of exogenous vs. endogenous vestibular challenges.

## Sensorimotor Orientation Challenges Faced by Astronauts and Balance Patients

Many of the problems space travelers must cope with are somewhat similar to those faced by certain types of vestibular patients (Buytaert et al., 2013). In fact, lack of vestibular function has been employed as an animal model for graviception in space (Jamon, 2014). It is likely that research into space adaptation syndrome could provide insights into clinical vestibular disorders such as vertigo (Clément and Ngo-Anh, 2013) and vice versa.

Table 1 lists several gross similarities between exogenous challenges to vestibular functioning caused by spaceflight (see Column B) and endogenous challenges caused by vestibular clinical pathology (Column C). The evidence for each Table 1 assertion is denoted via an alphabetical superscript linking to the published source.

**Table 1:** **Comparison of neurovestibular challenges and countermeasures (Column A) associated with reactions to spaceflight (Column B) vs. vestibular pathology (Column C)**.

(A) Challenges or countermeasures being compared	(B) Space	(C) Pathology
Head movements may trigger motion sickness (e.g., nausea, headache)	Yes^a,b,c,n^	Yes^o,q^
Head movements may trigger dizziness or vertigo	Yes^a^	Yes^1,o^
Head movements are voluntarily minimized during initial challenge	Yes^a^	Yes^o^
After the initial challenge, movement facilitates adaptation	Yes^a^	Yes^a,o^
The challenge causes decreased stability during standing and walking	Yes, after landing^a,c,d^	Yes^o,r^
Challenge is associated with cognitive and affective problems	Yes^b,d,e,f^	Yes^o,q,r,s^
Head movements may cause oscillopsia and decreased visual acuity	Sometimes^c,d,e^	Sometimes^o^
Challenge disrupts accurate perception of self-orientation and motion, especially during head movement	Sometimes^c,e^	Frequently^o,q^
Challenge can cause or reveal lateral asymmetries of function that trigger ocular torsion and disrupt estimates of visual vertical	Probably^c,g,h,n^	Sometimes^p^
Challenge can cause increased weighting or reliance on visual inputs	Yes, during/after flight^g,i,j,n^	Yes^o^
Challenge may disrupt work duties or activities of daily living	Yes^d,g^	Yes^2,o^
Anti-motion sickness medications have been employed	Yes^a,d^	Yes^q^
Adaptation/pre-adaptation has been employed as a countermeasure	Yes, before or after flight^g^	Yes, after deficit or surgery^t^
Sensorimotor compensation ranges from simple gain changes within a system to complicated substitution and learning mechanisms	Yes^k^	Yes^c^
Earth-referenced cues have been employed as assistance devices, countermeasures, or adjuncts to rehabilitation	Yes^c,d,k^	Yes^o^
Similar neurovestibular rehabilitation exercises aid recovery	Yes^d^	Yes^2,o^
Analogous challenges have been devised to simulate the problem	Yes^l,m^	Yes^2,u^

*Column B assertions are derived from Clément (2005)^a^, Lawson (2014a)^b^, Clément and Ngo-Anh (2013)^c^, Buckey (2006)^d^, Kanas and Manzey (2003)^e^, Porte and Morel (2012)^f^, Karmali and Shelhamer (2010)^g^, Buytaert et al. (2013)^h^, Clark and Rupert (1992)^i^, Taube et al. (2004)^j^, Mulavara et al. (2012)^k^, Moore et al. (2011)^l^, Dilda et al. (2011)^m^, and Lackner and Dizio (2006)^n^. Column C assertions are derived from Herman and Clendaniel (2014)^o^, Baloh and Honrubia (2001)^p^, Baloh and Halmagyi (1996)^q^, Brandt et al. (2014)^r^, Clément (2005)^a^, Lawson et al. (2013)^s^, Tjernström et al. (2009)^t^, and Grandizio et al. (2014)^u^. ^1^The type of response, activity, exercise, or challenge is similar to Column A but should not be inferred to be identical. ^2^One must also adapt in either case to changed somatosensory pressure cues, kinesthetic cues, and in a rotating room or space station, linear Coriolis and G-excess effects associated with moving along the radius of rotation*.

Neurovestibular structures function to provide a frame of reference for reflexive activities (e.g., gaze control relative to environment; head and body righting), and for perception of the body’s orientation and motion relative to the gravitational vertical. The many neurovestibular reactions and patterns of functioning mentioned in Table 1 are consistent with the inference that space adaptation and vestibular pathology each disrupt multiple reflexive and higher-order systems, including those responsible for perception, cognition, arousal, eye movement reflexes, and visceral functions. Neurovestibular adaptation to an exogenous challenge is, like endogenous vestibular pathology, more than a low-level reflexive process (Brandt et al., 2014). This is because the neurovestibular system (e.g., vestibular nucleus, cerebellar structures, etc.) constitutes a “system of systems,” i.e., a hub for coordinating, calibrating and integrating multiple adaptive processes among several neural systems (Balaban et al., 2012; Brandt et al., 2014; Shelhamer, 2015). While the underlying source of the challenges described in Table 1 differs for spaceflight vs. vestibular pathology (and in the case of pathology, is more difficult to identify using current tests), the fact remains that in either situation, the sensorimotor systems have been challenged to adapt and recalibrate. Whether the challenge is exogenous or endogenous in origin, it triggers many shared symptoms and orientation problems (prior to full adaptation). This overlap should stimulate researchers to consider the feasibility of employing similar approaches to facilitating adaptation. One example is provided below.

The essential neurovestibular aspects of the challenge represented by entry into microgravity can be most simply expressed as a mismatch between semicircular canal vs. otolith signals. After a lifelong tight coupling of canal-otolith information during head and body movement, astronauts in microgravity are subjected to a situation where the canal information is normal during head movement, but the otoliths are providing altered information from usual. This is analogous to many ground-based vestibular challenges that alter normal sensory integration. For example, on Earth, one is exposed to a canal-otolith mismatch when making head movements orthogonal to one’s body axis during prolonged (constant velocity) yaw rotation. In this case, the converse (of the spaceflight situation) occurs, in that the otoliths continue to provide normal signals, while the canal signals are altered. In either case, the mechanisms for central integration of canal and otolith information must adapt for normal coordination and well-being to be restored^1^.

**Figure 1:**
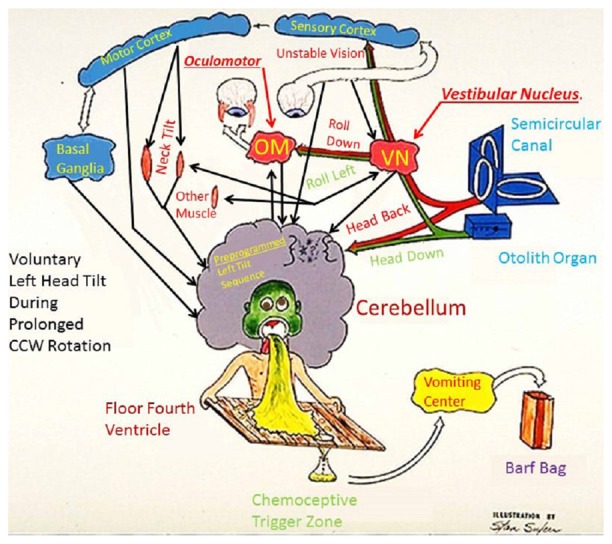
**Fred Guedry’s humorous model of the neural responses to a sensory rearrangement challenge.** The rearrangement is caused by Coriolis cross-coupled head movements employed as part of the adaptation challenge in Study #1 (Explanation in body of paper).

Figure 1 offers a simplified depiction of the neural response to a sensory rearrangement in the ground-based example of head movement during prolonged rotation^2^. If the subject is in the center of a room that is rotating counter-clockwise and he/she makes a left roll-tilt head movement, this yields a Coriolis cross-coupled signal (Guedry and Benson, 1978) from the canals which indicates head pitch backwards that acts in an axis orthogonal to the room rotation and the head movement. The appropriate reflex response to a fast head movement of this type would be a reflex movement (fast phase) of the eyes upward (If the movement is made slowly, then the eyes can respond sufficiently to avoid retinal slip and maintain visual acuity). Rapid head movement also induces an illusion of backward pitch velocity, because of a disagreement between the orientation of the angular impulse vector of the canal signal and the linear acceleration vector given by gravity (according to Guedry and Benson, 1978). If the stimulus is too strong for one’s adaptive capacity, sickness results (Graybiel, 1973). Slower head movements elicit subtler sensations and foster adaptation^3^. Repeated mild/slow head movements result in adaptation with the correct compensatory eye movements in response to roll head movement. Once adaptation has taken place, if the same head movement is made immediately upon reentering to the normal stationary environment, a negative aftereffect (i.e., an illusion of forward tilt) is experienced, leading to unnecessary postural compensation backwards, which could result in a fall backwards. Many past studies have corroborated the observation that adaptation to a rotating frame of reference induces negative aftereffects and postural incoordination upon returning to a normal environment (e.g., Graybiel et al., 1960; Fregly and Kennedy, 1965). We turn next to several plausible strategies for studying such environmental transitions and facilitating them.

## Plausible Strategies for Studying and Facilitating Sensorimotor Transitions, Especially Among Different Gravitoinertial Environments

Welch and Mohler (2014) and others described various sensorimotor and neurovestibular rearrangements, and stated general principles for facilitating adaptation to such rearrangements. We provide four examples of their adaptation principles here. The first principle is that adaptation is facilitated when the new sensorimotor rearrangement is consistent rather than continually changing. If the rearrangement is stable enough (i.e., consistent feedback is obtained moment-by-moment during exposure to a given stimulus), then adaptation occurs via interaction with the new arrangement, which generates immediate and reliable error- corrective feedback (e.g., concerning visual vs. haptic localization of a target). A second principle is that greater levels of adaptation can be reached with fewer unwanted symptoms by incremental exposure to the rearrangement (starting with a weak challenge) and by the use of distributed practice (i.e., optimal periods of exposure and rest). A third principle is that incremental and distributed exposure should be tailored to the susceptibility and adaptive capacity of the individual being adapted in order to avoid aversive conditioning, which would defeat the purpose of adaptation by instead amplifying the motion sickness response (Lawson, 2014a). Finally, since adaptation tends to be stimulus-specific, if one wishes to use one stimulus (e.g., rotation on Earth) to adapt someone to a different stimulus (e.g., microgravity), it is important to require the subject to adapt to a wide variety of relevant stimuli (i.e., multiple stable sensory rearrangement stimuli presented separately) in order to foster the ability of the central nervous system (CNS) to “learn to learn” and thereby confer generalizable protective adaptation (Wood et al., 2011). These are four of the general principles for facilitating adaptation and the factors which make adaptation occur readily or less readily. We consider next the specific case of space adaptation in regards to these principles.

During orbital spaceflight, the unloading of the otoliths by microgravity and their shifts related to head movements represent relatively stable sensory rearrangements that provide sufficient opportunities for error-corrective feedback so that adaptation can occur. Unfortunately, many of the other factors that foster optimal adaptation are not present in microgravity. For example, astronauts presently cannot be conveniently exposed to gradual increases in their duration of exposure to constant microgravity^4^, nor can they yet distribute their exposure during space missions by escaping weightlessness periodically while in orbit. Furthermore, all astronauts will be exposed to the same microgravity environment regardless of their susceptibility to space sickness, which means that more sensitive individuals may exceed their adaptive capacity and develop aversive conditioning. Finally, future astronauts who travel from the Earth to Mars and back again will be required to adapt to at least four different G transitions over the course of the voyage: (1) 1 G to 0 G during the voyage; (2) 0 G to 0.38 G on Mars; (3) 0.38 G to 0 G during the return voyage; and (4) 0 G to 1 G back on Earth (not counting the brief high-G phases of takeoff and reentry). Clearly, spaceflight lacks many of the features that would foster optimal acquisition of adaptation without the elicitation of adverse symptoms or effects.

The most direct means of facilitating adaptation during G transitions associated with space exploration would be to employ artificial gravity. For example, upon entry into microgravity, living most of the time onboard a space vehicle rotating at a fairly large radius to produce Earth’s 1 G would allow adaptation to 0 G operations to proceed more gradually and via distributed exposures. This strategy could also be used to gradually adapt an astronaut to the 0.38 G field of Mars prior to landing. However, it is not known precisely how much artificial gravity (i.e., positive G-magnitude, duration and frequency of exposure) would be needed to prevent bone and muscle loss under microgravity in orbit or 0 G in deep space. If daily exposure to 1 G is needed, then a gradual change of artificial gravity from 1 G to 0 G to 0.38 G in preparation for a Mars mission may not be advisable^5^. Furthermore, we have already discussed the adverse neurovestibular effects to be expected from movement inside a rotating frame of reference. The alternative of employing a brief period of regular exposure to a small centrifuge inside an otherwise weightless spacecraft has also been considered as an option for slowing bone and muscle atrophy (Young et al., 2009). The ultimate solution has not yet been settled for preventing bone and muscle atrophy while avoiding neurovestibular problems, but it is clear that any artificial gravity strategy that is adopted for a human mission to Mars will represent a complex trade-off among considerations such as cost, the need to make stable inflight astronomical observations, and human health and safety (Clément et al., 2016).

Are there ways in which space adaptation can be fostered without (or in addition to) artificial gravity? While gradual introduction to longer periods of spaceflight is not feasible prior to an actual mission, the magnitude of the initial challenge can be partially blunted by: (1) Medication—i.e., the use of medications that alleviate motion sickness without slowing adaptation; (2) Adaptation—programs of ground-based pre-adaptation to spaceflight; (3) Behavioral strategies—such as limiting the speed and amplitude of head movements during initial exposure to space; or (4) Sensory cueing—providing additional orientation cues to decrease the magnitude of the initial rearrangement, e.g., by enhancing somatosensory inputs to make the astronaut feel more grounded relative to the perceived “floor.” All four of these methods have been attempted, but most of them are not yet mature or fully understood. Medications provide some relief from nausea related to spaceflight, but some can be sedating and their effects on the process of adaptation vary (Wood et al., 1986). Ground-based generalized motion sickness adaptation has benefited aviators with airsickness (Acromite et al., 2010; Rogers and Van Syoc, 2011) and ground programs have been developed for astronauts as well. This approach is in accord with the general “learning to learn” adaptation principle described above, but it is labor-intensive and adaptation must be maintained without a lengthy period of layoff from exposure.

Behavioral strategies are often attempted in situations where people cannot escape a nauseogenic stimulus. In space, during parabolic flight sorties, or during military air/sea operations, it is common to see people restricting their head movements initially, and then later resuming normal head movements once they feel better. This strategy tends to minimize vestibular and cervical activity, allowing passengers to initially limit (and then gradually increase) their exposure to that part of the challenging stimulus which is under their control. In space, limiting one’s head movements means one is exposed only to the relatively static aspect of the vestibular challenge caused by the unloading of the otolith crystals, rather than also being exposed to the dynamic challenge associated with nonterrestrial movements of the otolith crystals during head movements under weightless conditions. Restricting head movement is a useful strategy for limiting the nauseogenic challenge. Unfortunately, since many astronaut and military duties must be performed quickly and require frequent head movement, this strategy can be applied less successfully in such occupations than it can in a less-demanding setting, such as during a pleasure cruise at sea. Furthermore, while astronauts and vestibular patients suffering from nausea or vertigo may prefer to lie quietly with their eyes closed, such behaviors could become counterproductive habits that limit beneficial adaptation. Vestibular patients often limit their activity when they should engage in movement in order to recover (Herdman, 1997). In fact, a key principle of vestibular rehabilitation is that patients should always move up to the limits of their ability and seek to extend those limits. Herdman recommends making exercises steadily more difficult in various ways, such as incorporating head movements while balancing. Making head movements while balancing should also be beneficial for astronaut testing and readaptation, if applied judiciously^6^ (Clément, 2005; Paloski et al., 2006; Jain et al., 2010). When head movements are executed as part of readaptation, it is likely that additional somatosensory information concerning the true orientation of the Earth gravitational vertical will benefit the readaptation process. Below, we discuss the benefits of enhancing somatosensory input in space and upon return to Earth.

The final strategy for helping astronauts with space adaptation syndrome (or readaptation to Earth) is to provide additional orientation cues to decrease the magnitude of the initial rearrangement, by enhancing somatosensory inputs to make the astronaut feel more grounded relative to the perceived “floor” or other significant frames of reference. It has long been known that situations where the locally-defined “up and down” are ambiguous or unexpected can elicit disorientation and motion sickness symptoms among astronauts (Nicogossian et al., 1988). It is also known that providing a tactile reference can aid with orientation and performance during spaceflight (Lackner and DiZio, 2000; van Erp and van Veen, 2006; Clément et al., 2007) or piloting of an aircraft (Rupert, 2000; Kelley et al., 2013; Brill et al., 2014). In these cases, body tilt or motion that has not been perceived correctly causes the tactile cue to vibrate much like a rumble strip vibrates a driver who is veering off the road. For example, if the astronaut starts to tilt right, the body movement is detected by a sensitive posturography platform or body-worn accelerometers, causing the tactile belt to activate on the right side to warn him or her. It is likely that such feedback about one’s body orientation and motion will foster sensorimotor adaptation following a vestibular challenge as well (Lawson and Rupert, 2010).

It is widely accepted in neuroscience that the brain exhibits adaptive plasticity under many kinds of sensorimotor challenges and that “neurons that fire together wire together.” It is clear that recovery of balance (and gaze) control after challenges such as labyrinthine concussion is largely a process of neurological adaptation based on sensory feedback. One of the key principles of vestibular rehabilitation is that movement, and the associated sensory feedback from movement, are critical for such adaptation. This fact is vividly illustrated by the large deleterious effect of restricted mobility on the rate of clinical recovery of baboons following unilateral transaction of the vestibular nerve (Herdman, 1997). Unstructured movement is not ideal, however. There are several experiments demonstrating the benefits of specific vestibular rehabilitation training (Horak et al., 1992; Herdman, 1996). Such studies have found that: (1) patients who did vestibular exercises showed greater balance improvement than patients on a general conditioning program (or vestibular suppressant medication); (2) ambulation plus vestibular exercises led to better postural stability and less disequilibrium than ambulation alone.

The choice of an optimal sensory modality for sway-feedback is not trivial. A visual feedback device is simple to program and display and is readily available from various manufacturers. However, if visual cueing technology is eventually made wearable not only for convenient/portable adaptation training, but also for non-invasive sensory prosthetic applications (e.g., “orienting glasses” that show the “true horizon”), the display would produce light cues for orientation which interfere with one’s retinal sensitivity for natural, ambient visual environmental orientation cues in low light situations. Thus, balance cues would be enhanced by a visual prosthesis, but natural cues for avoiding collisions would be degraded even further than they are by existing head injuries or the natural aging process (Kennedy et al., 2001). Even in daylight, attention would be split continually between the visual sway cue and other important tasks dependent upon vision. Some of the important natural functions of vision could be disrupted, such as perception of distant objects, target localization, and interpretation of symbolic information (Lawson, 2014b).

Conversely, tactile sway feedback is well-suited for balance assistance or rehabilitation applications, since it is relatively intuitive and less distracting to visual processing than an additional visual display (Rupert, 2000). Tactile sensations must be processed quickly and intuitively during standing and locomotion, because they are so essential to survival. People born without vision or hearing commonly live their full life span, but individuals born without a sense of touch do not usually survive. Brain development and anatomy reflect the role of touch as a primary survival sense (Rupert, 1998). For example, large regions of the somatosensory homunculus are devoted to the hands, feet, and face. Moreover, within the midbrain, the sensory systems are topologically arranged to map to the external environment, with the tactile system in the lowest layer, while auditory and visual representations of the world are sequentially overlaid upon it during development. Visual and auditory connections and architecture are built on the base architecture established by the sense of touch. The other sensory systems depend on touch for development, and the midbrain is the lowest level that can be stimulated to elicit a coordinated orientation response of eyes, ears, head, neck, and torso moving to attend to a point in space (Bisti et al., 1974; Abrahams and Rose, 1975). For these and other reasons (Rupert and Lawson, 2010), touch is a good choice for orientation cueing and sway feedback. Therefore, we have chosen vibrotactile inputs as one of the sway-cueing techniques that should be incorporated into balance rehabilitation (Lawson et al., 2012).

There is evidence that enhancing tactile sensory feedback concerning one’s orientation can be beneficial in the clinical setting to prevent falling and aid with balance rehabilitation. Use of a cane by patients with peripheral neuropathy reduces their risk of losing balance on unstable surfaces, especially under low-light conditions (Ashton-Miller et al., 1996). The cane is not simply for support, since blind people find that light touch contact of a cane is as effective as forceful contact in reducing their postural sway (Jeka et al., 1996). In fact, light touch and kinesthesia derived from holding one’s finger on a stable reference bar reduces sway as much as visual cues, even in the absence of enough contact force to provide support (Holden et al., 1994; Jeka and Lackner, 1994). Similarly, somatosensory feedback concerning one’s true rotation can diminish the disorientation and sickness associated with Coriolis cross-coupling (Guedry, 1978; Lawson et al., 1997). Somatosensory feedback is also useful for avoiding spatial disorientation during aviation. A matrix or an array of vibratory touch signals to the torso kept aviators oriented during in-flight tests we conducted (Rupert et al., 1994; Rupert, 2000; McGrath et al., 2004). Finally, a vibrotactile array has been used in various orientation cueing devices for reducing sway (Wall et al., 2001; Lawson et al., 2012).

We turn next to a brief review of two preliminary studies that are relevant to the elicitation, assessment and mitigation of exogenous or endogenous vestibular adaptation problems (Table 2). The first study (McGrath et al., 1993) explored exogenous ground-based gravitoinertial force alteration and balance-testing strategies that might prove useful as proxies or assessments of balance deficits caused by vestibular maladies or aftereffects of spaceflight. The second study (Atkins and Gottshall, 2014) concerned a sensory cueing device (and associated strategy) that had been used to improve in-flight spatial orientation and was later explored as a way to improve balance recovery following endogenous vestibular pathology. Below, we provide a brief review of these two exploratory studies.

**Table 2:** **The two preliminary studies briefly reviewed in this article**.

Study	Study 1 (McGrath et al., 1993):	Study 2 (Atkins and Gottshall, 2014):
	Effects of adaption to a neurovestibular challenge upon standing balance	Tactile cueing effect on vestibular rehabilitation of balance patients
Question	Can the aftereffects of an exogenous ground-based vestibular challenge disrupt balance?	Can enhanced Earth-referenced sensory cueing stabilize balance following an endogenous vestibular challenge/alteration?
Participants	32 healthy military aviator candidates	25 elderly balance patients
Finding	Balance was worse after exposure to a vestibular challenge	Balance rehabilitation was improved
Space application	Ground-based proxy for space adaptation	Adjunct to balance readaptation training after landing

## Brief Highlights from Pilot Study 1 Regarding Postural Aftereffects of an Exogenous Vestibular Adaptation Challenge

This preliminary research (McGrath et al., 1993; Shepard et al., 1998) explored a ground-based vestibular challenge meant to disrupt balance transiently. The subjects were 32 healthy military aviator candidates, all of whom had passed a naval flight physical examination with no history or indication of vestibular disorders. They were exposed to systematic head and body movements inside a rotating room (Hixson and Anderson, 1966) in order to assess adaptation-related balance aftereffects associated with this altered gravitoinertial force environment. While the room rotated slowly in the yaw axis, the subjects made a systematic series of head and locomotory movements within the room. Under these circumstances, voluntary movement yields mild Coriolis cross-coupling sensations (Guedry and Benson, 1978), while locomotion yields vestibular and somatosensory (skin, muscle, and joint) signals that are at variance with visual information and with intended movement trajectories and associated reafference (Roy and Cullen, 2001). This results in adaptation to the rotating frame of reference but induces postural incoordination upon returning to a stationary environment (Graybiel et al., 1960; Fregly and Kennedy, 1965). Immediately upon exiting the rotating room, the subjects performed a challenging version of the Neurocom Equitest Sensory Organization Test #5 (SOT5; NeuroCom International, Inc., 2007) that is appropriate for high-performing individuals such as military aviator candidates. In this test, subjects closed their eyes and moved their heads while standing on a platform that measures their center-of-gravity and tilts the platform in the direction the center-of-gravity is shifting. This challenging test robs subjects of most of the useful balance feedback they would usually get from vision, leg kinesthesia, and foot contact, and forces them to rely upon the CNS interpretation of changing vestibular information to locate the direction of gravity.

**Figure 2:**
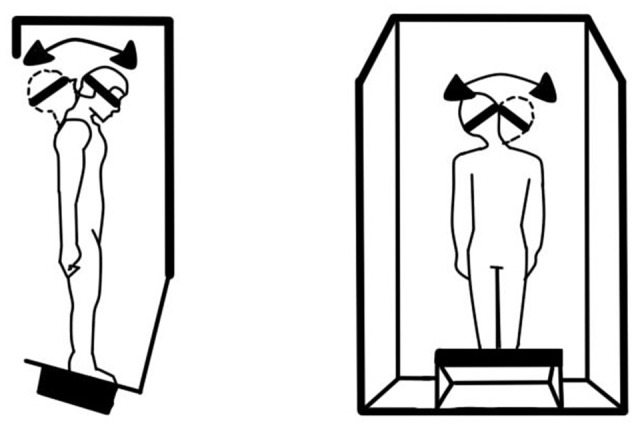
**Schematic of the modified Sensory Organization Test 5 (SOT5) explored in Study #2, which required the subject to make head movements while balancing without helpful cues from vision (eyes closed) or ankle kinesthesia (unstable platform)**.

Following the rotation period, the subject completed two sessions (of three trials each) of the balance test. After only 10 min of adaptation to the rotating room, the subjects had already adapted sufficiently to exhibit observable vestibulo-ocular reflexes and coordinated walking movements appropriate to the new rotating environment. This is a striking example of adaptive plasticity allowing rapid adjustment to a complex stimulus. The balance testing situation is shown in Figure 2.

**Figure 3:**
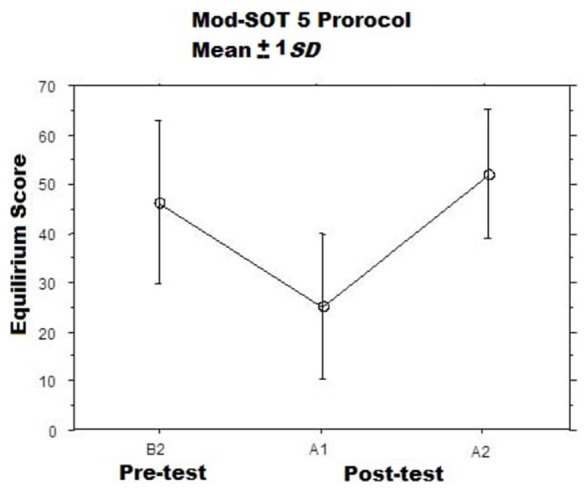
**Mean modified SOT5 Equilibrium Scores Before (B2) and at two intervals After (A1, A2) the adaptation sessions (McGrath et al., 1993).** The A1 assessments were commenced immediately after the adaptation session ended, while the A2 assessments were commenced approximately 15 min after the A1.

When subjects left the room to be tested on the balance platform, they showed significantly worse post-rotation balance performance during the modified SOT5 involving balancing while moving one’s head^7^. We observed large variability in performance but detected a significant difference between the mean baseline (pre-rotation) balance score vs. the first post-rotation test score (*Wilcoxon Signed-Rank Test: p* < 0.05; Figure 3). As planned, the vestibular insult was transient: balance performance had returned sufficiently to normal by the end of the second post-testing session for no further difference to be detected vs. baseline.

Measures of sway are interesting for sensitively quantifying deficits in standing balance, but the critical functional indicator of instability among patients and astronauts is falling. Falling was common during the first post-test, occurring among 25 (of the 32) subjects on the first trial of the first post-testing session. This suggests that the adaptation protocol created a profound aftereffect. As desired, the falling aftereffect was temporary and readaptation to normal conditions was rapid. This statement is supported by the fact that only five subjects fell during all three trials of the second post-testing session. In summary, the ground-based adaptation task worked as planned, inducing a temporary and reversible vestibular insult with functional consequences for standing balance detectible during a challenging balance task. As with imbalance following spaceflight, the elicited balance deficit was attributable to the aftereffects of adaptation to an unusual gravitoinertial force environment.

The addition of head movements to SOT5 was an interesting feature of this pilot study (McGrath et al., 1993; Shepard et al., 1998), and one which has promise for space testing applications. The execution of voluntary head movements while standing required CNS interpretation of complex vestibular and cervical inputs during an already difficult balancing task (SOT5). This challenging testing approach has already been implemented successfully by others, for the testing of high-functioning individuals. For example, standing while performing head movements has been used successfully by NASA as an objective test to determine when returning astronauts are safe to fly (Paloski et al., 2006). Similarly, Neurocom has added a capability for head movements during their Equitest protocol (Jain et al., 2010).

During the execution of Study #1 (McGrath et al., 1993; Shepard et al., 1998) concerning the aftereffects of adaptation to a rotating frame of reference, we happened to have tactile orientation cueing devices readily available from our aviation projects, so we performed a small, informal pilot test of tactile balance cueing. We had demonstrated in Study #1 that adaptation to a rotating environment could be used to create a temporary, rapidly-reversible vestibular insult which caused imbalance. In our informal tactile cueing study, we placed tactors on five rotating-room-adapted subjects and then provided center-of-gravity information from the platform to the front or back of their torsos to indicate which way they were swaying. We qualitatively observed that the subjects could all maintain upright posture much better with tactile cueing than without it. This initial pilot test needs to be confirmed by a full study, but the indications from subsequent studies (described in Rupert and Lawson, 2010, and Lawson et al., 2012) are that tactile cueing can be used intuitively to improve balance performance in balance-compromised individuals. Such findings justified a government Small Business Innovative Research (SBIR) program to support further development of tactile cueing balance systems, one of which is described briefly in Study #2, below.

## Brief Highlights from Pilot Study 2 Regarding Benefits of Tactile Cueing during Adaptive Recovery from an Endogenous Vestibular Challenge

**Figure 4:**
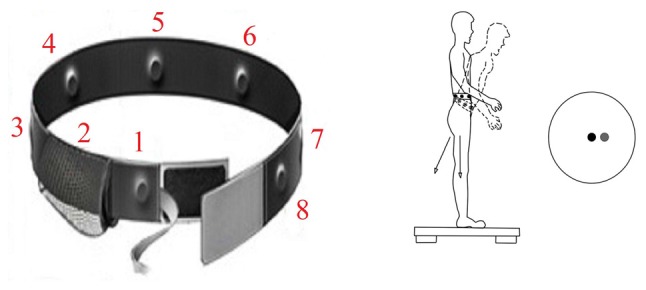
**At left is a version of the sway cueing belt, which supplies vibrotactile warnings of sway in eight directions.** As the center of gravity moves forward, the forward tactor is activated similar to a rumble strip alerting a car driver when he is running off the road. At right is a schematic of forward sway of the body away from center of sway platform pressure (black circle) which triggers activation of front tactor (relative direction of sway motion warning indicated by the gray circle).

We wished to determine whether enhanced vibrotactile sway tactile cueing (Figure 4) could improve balance among vestibular patients or augment their treatment outcomes during vestibular rehabilitation (Hastings-Atkins, 2010; Rupert and Lawson, 2010; Atkins and Gottshall, 2014; Lawson et al., 2014). Therapy included a maximum of 12 physical therapy intervention sessions over 6 weeks^8^.

Twenty-five elderly balance rehabilitation patients participated in a preliminary study (Atkins and Gottshall, 2014; Rupert, 2016). Aging is associated with increased imbalance, due to a decrease in vestibular hair cells, vestibular neurons, and otoconia, as well as a decline in neural compensation for latent, subclinical vestibular conditions (Herman and Clendaniel, 2014). This pilot study compared the outcomes of standard physical therapy plus the tactile cueing device/strategy vs. standard physical therapy alone. Sway was measured via the regular Equitest SOT battery (including the vestibularly-demanding SOT5^9^), and clinical outcomes of vestibular rehabilitation were tracked before therapy and four times during therapy. The treatment outcomes of therapy were estimated by changes in the number of falls during SOT5 testing, and by Berg Balance Scale (BBS) changes in one’s fall risk (41–56 = low risk category, 21–40 = medium, or 0–20 = high; Muir et al., 2008).

Both groups reached the same sway improvement eventually, with the group in the enhanced tactile feedback condition (*n* = 14) showing a nonsignificant trend for earlier improvement of balance performance scores during computerized posturography compared to subjects who received standard-of-care physical therapy (*n* = 11). Calculations of percent-of-change in mean composite scores on the SOT (#1–6) determined that the Device group realized maximum improvement after eight sessions. Controls reached maximum improvement after 12 sessions (by which time both groups were performing similarly).

**Figure 5:**
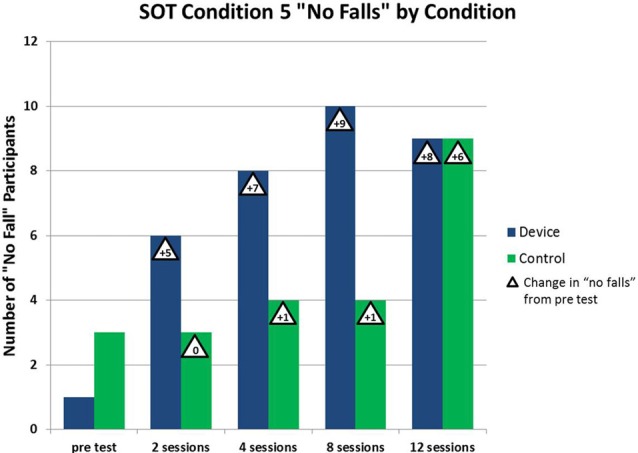
**Beneficial effect of vestibular rehabilitation.** As the number of vestibular rehabilitation sessions increases, the number of patients who do not fall during SOT #5 increases. The beneficial effect is more pronounced with the device group using the tactile cue (Atkins and Gottshall, 2014).

Regarding estimates of the clinical effectiveness of the therapy, we observed better avoidance of falling in the Device group. In the Device group, the number of participants who did not fall during vestibularly-demanding balance testing (SOT5) increased by five from pre-testing vs. testing after two sessions of enhanced tactile cueing device therapy, whereas the number of Control group (standard of care therapy) participants who did not fall did not change at all over the same period (see Figure 5)^10^. Similarly, of the 12 subjects in this study who were initially placed in the high or medium fall risk categories (according to the well-established BBS), all of those in the device/treatment group reduced their fall risk category after only two sessions of physical therapy, whereas only 43% of those in the control group did so by that time. In summary, the enhanced tactile sway cue appeared to foster better balance rehabilitation outcomes by making falling less likely. This pilot test needs to be confirmed by a full study, however. Also, we recommend evaluations using astronauts recovering from the effects of spaceflight.

## Discussion

Balance testing and assistance efforts originally intended to benefit military aviators or balance patients are yielding cross-disciplinary benefits for astronauts as well (Rupert, 2000; Wall et al., 2001; McGrath et al., 2004; Wall and Kentala, 2005; Peterka et al., 2006; Hastings-Atkins, 2010; Rupert and Lawson, 2010; Rupert et al., 2011; Atkins and Gottshall, 2014; Lawson et al., 2014; Rupert, 2016). It appears that healthy military aviator candidates (a population highly relevant to the astronaut corps) respond to a ground-based sensorimotor challenge^11^ to their spatial orientation functioning by adapting to that challenge and then exhibiting an adaptation aftereffect upon reentry into the normal gravitoinertial force environment. This situation is analogous to the sensorimotor challenge of microgravity, which requires adaptation of an astronaut’s acceleration-sensing systems and, once adaptation is completed, the astronaut’s balance is disrupted upon reentry into the terrestrial 1g environment. The process of neurovestibular adaptation to a ground-based or space-based alteration of the gravitoinertial environment also shares some similarities to the challenge faced by a patient seeking to recover from a vestibular pathology. It is likely that astronauts returning from space can benefit from many of the vestibular rehabilitation exercises and sensory cueing strategies employed on balance patients, possibly with enhanced tactile sway feedback as a feature of therapy. We recommend that this balance rehabilitation strategy undergoes further evaluation and validation using clinical and astronaut populations. Some modifications of the rehabilitation exercise and cueing strategies may be needed to fit the particular needs of these two populations, who will tend to differ in their mean ability to adapt rapidly. An elderly vestibular patient may find some exercises difficult that a younger astronaut would find easy. For example, Gottshall and colleagues usually employ the Functional Gait Assessment studying balance problems among relatively young military personnel, rather than similar but less challenging gait tests (e.g., Atkins and Gottshall, 2014). Similarly, the illusory perceptions which accompany head movement following spaceflight (e.g., direction of perceived tilt) will not be identical to those accompanying head movement following clinical vestibular pathology, so the type of helpful cue will need to be tailored to the situation. In general, if any differences in these two populations exist in their ability to benefit from tactile balance cues, we would expect the astronauts to derive benefit more readily than the clinical vestibular patients, due to the fact that their vestibular systems are intact (rather than injured endogenously), their average level of fitness is higher, their average age is younger, and their average intelligence and persistence (for rehabilitation, etc.) is higher. Finally, the astronauts tend to experience episodes of vertigo/imbalance that are relatively predictable (compared to the varied balance effects caused by the many different types of vestibular pathology).

In addition, continued improvement of tactile displays is needed beyond the simple eight-tactor belt on/off directional cues that have been employed in the past to inform a person that he/she has reached a designated sway limit. Recent technological developments have yielded smaller, lighter vibrotactors with control units that can drive a large array of tactors. This approach would more closely approximate the thousands of tactile receptors that nature has provided to assist normal terrestrial movement. Such advanced arrays could provide a high-resolution, dynamic tactile display to convey tactile flow over the surface of the body in ways that would aid the accurate perception of self-motion, orientation, and closing with significant objects (Lawson, 2014b). They should also decrease the severity of adverse symptoms associated with sensory rearrangements such as those caused by entry into an unusual gravitoinertial force environment.

A final consideration for the transition of any innovation is whether the targeted users of the new tool or strategy will accept and use it. Fortunately, physical therapists to whom we have demonstrated the device have told us the tactile balance rehabilitation device is user-friendly and desirable. More importantly, the tactile rehabilitation device has been incorporated successfully as an available treatment modality during regular practice at two physical therapy clinics to date (at the Naval Medical Center San Diego and the Defence Medical Rehabilitation Centre Headley Court). The device is going to market soon, so improved, widespread evaluations should be forthcoming from multiple clinics.

## Author Contributions

BDL: led the writing effort, assisted with the studies reviewed, assisted with development of apparatus. AHR: contributed to the writing effort, initiated studies 1 and 2, assisted with development of apparatus. BJM: contributed to analyses, led study 1, developed apparatus.

## Funding

Studies #1 and #2 described herein were primarily sponsored by the Naval Air Systems Command and the U.S. Army Medical Research and Material Command, respectively.

## Conflict of Interest Statement

The authors declare that the research was conducted in the absence of any commercial or financial relationships that could be construed as a potential conflict of interest.

## Footnotes

^1^One must also adapt in either case to changed somatosensory pressure cues, kinesthetic cues, and in a rotating room or space station, linear Coriolis and G-excess effects associated with moving along the radius of rotation.

^2^Neurovestibular researchers will be interested to learn that the late Fred Guedry conceived of the figure and did the initial sketch for the final, light-hearted cartoon shown in Figure 1, which he displayed in his office.

^3^The sensation is dependent also on the amplitude of head movement and the velocity of room rotation.

^4^It is possible that repeated exposure to parabolic flight could confer some resistance to microgravity but this entails a changing stimulus with brief periods of weightlessness alternating with high G.

^5^Some small centrifuge countermeasures protocols call for brief exposures to even greater than 1 G.

^6^Head movements should be slow and small at first to avoid adverse symptoms, then gradually increased in speed, magnitude and frequency as adaptation is acquired.

^7^The standard SOT#5 test (without head movements) was not sensitive to the effects of rotating room adaptation.

^8^Therapy ended at 12 sessions or upon normalization of Sensory Organization Test scores, whichever occurred first.

^9^Only 5 of the 25 patients felt able to comfortably execute the modified SOT5 requiring head movements, so modified SOT5 data were not analyzed.

^10^The treatment group had one “no fall” during pre-testing vs. six “no falls” following two sessions of therapy. Conversely, the control group had three “no falls” during pre-testing and three “no falls” after two sessions of therapy.

^11^This ground-based challenge exists only in Star City (Moscow) and at Brandies University (Ashton Graybiel Laboratory, Waltham, MA, USA) presently. As plans for a human landing on Mars reach maturity (and G-transitions and artificial gravity become important topics again), this paradigm should become as important as it was during the 1960s Navystudies done by Graybiel et al. (1960) on the Slow Rotation Room and the Coriolis Acceleration Platform in Pensacola, FL, USA.

## References

[B1] Abrahams V. C. Rose P. K. (1975). Projections of extraocular, neck muscle and retinal afferents to superior colliculus in the cat: their connections to cells of origin of tectospinal tract. J. Neurophysiol. 38, 10–18. 162939 10.1152/jn.1975.38.1.10

[B2] Acromite M. T. Cowings P. Toscano W. Carl D. Porter H. O. (2010). “NASA-Navy telemedicine: autogenic feedback training exercises for motion sickness,” in 81st Annual Scientific Meeting Aerospace Medical Association (Phoenix, AZ: NASA Technical Reports Server), 9–13.

[B3] Ashton-Miller J. A. Yeh M. W. Richardson J. K. Galloway T. (1996). A cane reduces loss of balance in patients with peripheral neuropathy: results from a challenging unipedal balance test. Arch. Phys. Med. Rehabil. 77, 446–452. 10.1016/s0003-9993(96)90032-5 8629920

[B4] Atkins K. Gottshall K. (2014). “Treatment of vestibular/balance dysfunction via multimodal vibrotactile, visual and auditory feedback,” in Abstracts from the 28th Bárány Society Meeting 25–28 May, 2014 (Buenos Aires, Argentina: Journal of Vestibular Research), 24, 130.

[B5] Balaban C. D. Hoffer M. E. Gottshall K. R. (2012). Top-down approach to vestibular compensation: translational lessons from vestibular rehabilitation. Brain Res. 1482, 101–111. 10.1016/j.brainres.2012.08.040 22981400 PMC3490401

[B6] Baloh R. W. Halmagyi G. M. (1996). Disorders of the Vestibular System. New York, NY: Oxford University Press.

[B7] Baloh R. W. Honrubia V. (2001). Clinical Neurophysiology of the Vestibular System. 3rd Edn. New York, NY: Oxford University Press.

[B8] Bisti S. Maffei L. Piccolino M. (1974). Visuovestibular interactions in the cat superior colliculus. J. Neurophysiol. 37, 146–155. 4811972 10.1152/jn.1974.37.1.146

[B9] Brill J. C. Rupert A. H. Lawson B. D. (2014). “Tactile situation awareness system (TSAS) as a compensatory aid for sensory loss,” in Proceedings of the Human Factors and Ergonomics Society 58th Annual Meeting (Chicago, IL), 27–31. October, 1028–1032. Paper number 222_589.

[B10] Brandt T. Strupp M. Dieterich M. (2014). Towards a concept of disorders of “higher vestibular function”. Front. Integr. Neurosci. 8:47. 10.3389/fnint.2014.00047 24917796 PMC4041089

[B11] Buckey J. C. (2006). Space Physiology. New York, NY: Oxford University Press.

[B12] Buytaert K. I. MacDougall H. G. Moore S. T. Clement G. Pattyn N. Migeotte P. F.. (2013). Validation of centrifugation as a countermeasure for otolith deconditioning during spaceflight: preliminary data of the ESA SPIN study. J. Vestib. Res. 23, 23–31. 10.3233/VES-130469 23549052

[B13] Clark J. B. Rupert A. H. (1992). Spatial disorientation and dysfunction of orientation/equilibrium reflexes: aeromedical evaluation and considerations. Aviat. Space Environ. Med. 63, 914–918. 1417656

[B14] Clément G. (2005). Fundamentals of Space Medicine. New York, NY: Springer. The Space Technology Library.

[B15] Clément G. Arnesen T. N. Olsen M. H. Sylvestre B. (2007). Perception of longitudinal body axis in microgravity during parabolic flight. Neurosci. Lett. 413, 150–153. 10.1016/j.neulet.2006.11.047 17174031

[B16] Clément G. R. Charles J. B. Paloski W. H. (2016). Revisiting the needs for artificial gravity during deep space missions. Rev. Hum. Space Explor. 1, 1–10. 10.1016/j.reach.2016.01.001

[B17] Clément G. Ngo-Anh J. T. (2013). Space physiology II: adaptation of the central nervous system to spaceflight—past, current and future studies. Eur. J. Appl. Physiol. 113, 1655–1672. 10.1007/s00421-012-2509-3 23053128

[B18] Dilda V. MacDougall H. G. Moore S. T. (2011). Tolerance to extended galvanic vestibular stimulation: optimal exposure for astronaut training. Aviat. Space Environ. Med. 82, 770–774. 10.3357/asem.3051.2011 21853854

[B19] Fregly A. R. Kennedy R. S. (1965). Comparative effects of prolonged rotation at 10 rpm on postural equilibrium in vestibular normal and vestibular defective human Subjects. Res. Rep. U S Nav. Sch. Aviat Med. 15, 1–21. 14313057

[B740] Grandizio C Lawson B. King M. Cruz P. Kelley A. Erickson B. (2014). *Development of a Fitness-for-Duty Assessment Battery for Recovering Dismounted Warriors*. (USAARL Technical Report No. 2014-18). *USAARL Technical Report No. 2014-18*. (Fort Rucker, AL: U.S. Army Aeromedical Research Laboratory).

[B20] Graybiel A. (1973). “The vestibular system,” in Bioastronautics Data Book, eds Parker J. F. West V. R. (Washington, DC: NASA), 533–609.

[B21] Graybiel A. Clark B. Zarriello J. J. (1960). Observations on human subjects living in a “Slow Rotation Room” for periods of two days. Arch. Neurol. 3, 55–73. 10.1001/archneur.1960.00450010055006 13829134

[B22] Guedry F. E. (1978). Visual counteraction of nauseogenic and disorienting effects of some whole-body motions: a proposed mechanism. Aviat. Space Environ. Med. 49, 36–41. 304720

[B23] Guedry F. E. Benson A. J. (1978). Coriolis cross-coupling effects: disorienting and nauseogenic or not? Aviat. Space Environ. Med. 49, 29–35. 304719

[B24] Hastings-Atkins K. L. (2010). Vibrotactile postural control in patients that have sit-to-stand balance deficit and fall. USAARL Technical Report, Report No. USAARL-2010-18.

[B25] Herdman S. J. (1996). “Vestibular rehabilitation,” in Disorders of the Vestibular System, eds Baloh R. W. Halmagyi G. M. (New York, NY: Oxford Press), 583–598.

[B26] Herdman S. J. (1997). “Balance rehabilitation: background, techniques and usefulness,” in Handbook of Balance Function Testing, eds Jacobson G. P. Newman C. W. Kartush J. M. (New York, NY: Thompson), 392–406.

[B27] Herman S. J. Clendaniel H. A. (2014). Vestibular Rehabilitation. (4th Edn) Philadelphia, PA: F.A. Davis Company.

[B75] Hixson W. C. Anderson J. J. (1966). The Coriolis Acceleration Platform: A Unique Vestibular Research Device. Virginia: The Defense Technical Information Center.

[B28] Holden M. Ventura J. Lackner J. R. (1994). Stabilization of posture by precision contact of the index finger. J. Vestib. Res. 4, 285–301. 7921347

[B29] Horak F. B. Nutt J. G. Nashner L. M. (1992). Postural inflexibility in Parkinsonian subjects. J. Neurol. Sci. 111, 46–58. 10.1016/0022-510x(92)90111-w 1402997

[B30] Jain V. Wood S. J. Feiveson A. H. Black F. O. Paloski W. H. (2010). Diagnostic accuracy of dynamic posturography testing after short-duration spaceflight. Aviat. Space Environ. Med. 81, 625–631. 10.3357/asem.2710.2010 20597240

[B31] Jamon M. (2014). The development of vestibular system and related functions in mammals: impact of gravity. Front. Integr. Neurosci. 8:11. 10.3389/fnint.2014.00011 24570658 PMC3916785

[B32] Jeka J. J. Easton R. D. Bentzen B. L. Lackner J. R. (1996). Haptic cues for orientation and postural control. Percept. Psychophys. 58, 409–423. 10.3758/bf03206817 8935902

[B33] Jeka J. J. Lackner J. R. (1994). Fingertip contact influences human postural control. Exp. Brain Res. 79, 495–502. 10.1007/bf00229188 7813685

[B34] Kanas N. Manzey D. (2003). Space Psychology and Psychiatry. (Vol. 16), New York, NY: Springer.

[B35] Karmali F. Shelhamer M. (2010). Neurovestibular considerations for sub-orbital spaceflight: a framework for future investigation. J. Vestib. Res. 20, 31–43. 10.3233/VES-2010-0349 20555165 PMC3634569

[B36] Kelley A. M. Cheung R. Lawson B. D. Rath E. Chiasson C. Ramiccio J. G.. (2013). Tactile cues for orienting pilots during hover over moving targets. Aviat. Space Environ. Med. 84, 1255–1261. 10.3357/asem.3669.2013 24459796

[B37] Kennedy R. S. Jentsch F. Smither J. A. (2001). “Looming detection among drivers of different ages,” in Proceedings of the Human Factors and Ergonomics Society 45th Annual Meeting (Santa Monica, CA: Human Factors and Ergonomics Society), 240–244.

[B38] Lackner J. R. DiZio P. (2000). Human orientation and movement control in weightless and artificial gravity environments. Exp. Brain Res. 130, 2–26. 10.1007/s002210050002 10638437

[B39] Lackner J. R. Dizio P. (2006). Space motion sickness. Exp. Brain Res. 175, 377–399. 10.1007/s00221-006-0697-y 17021896

[B40] Lawson B. D. (2014a). “Motion sickness symptomatology and origins,” in Handbook of Virtual Environments: Design, Implementation and Applications, 2nd Edn., eds Hale K. S. Stanney K. M. (New York, NY: CRC Press, an imprint of Taylor & Francis Group, LLC), 531–600.

[B41] Lawson B. D. (2014b). “Tactile displays for cueing self-motion and looming: what would Gibson think?,” in Proceedings of the 5th International Conference on Applied Human Factors and Ergonomics, eds Ahram T. Karwoski W. Marek T. (Kraków), 928–938.

[B42] Lawson B. D. Rupert A. H. (2010). “Vestibular aspects of head injury and recommendations for evaluation and rehabilitation following exposure to severe changes in head velocity or ambient pressure,” in Peer-Reviewed Proceedings of the International Conference on Human Performance at Sea (HPAS), eds Turan O. Bos J. Stark J. Colwell J. (Glasgow, UK: University of Strathclyde), 367–380.

[B43] Lawson B. D. Rupert A. H. Guedry F. E. Grissett J. D. Mead A. M. (1997). “The human-machine interface challenges of using virtual environment (VE) displays aboard centrifuge devices,” in Proceedings of the 7th International Conference on Human-Computer Interaction (San Francisco, CA), 945–948.

[B44] Lawson B. D. Rupert A. H. Kelley A. M. (2013). Mental disorders comorbid with vestibular pathology. Psychiatr. Ann. 43, 324–327. 10.3928/00485713-20130703-07

[B45] Lawson B. D. Rupert A. H. Legan S. (2012). Vestibular balance deficits following head injury: recommendations concerning evaluation and rehabilitation in the military setting. USAARL Technical Report, Report No. 2012-10.

[B46] Lawson B. D. Rupert A. H. Raj A. K. Parker J. N. Greskovich C. (2014). Invited lectures from a spatial orientation symposium in honor of Frederick Guedry, Day 1. USAARL Technical Report, Report No. 2014-10.

[B47] McGrath B. J. Estrada A. Braithwaite M. G. Raj A. K. Rupert A. H. (2004). Tactile Situation Awareness System flight demonstration. USAARL Technical Report, Report number 2004-10.

[B48] McGrath B. J. Rupert A. H. Ruck R. (1993). “Postural equilibrium testing of aviators: evaluation of the Neurocom Equitest system using a modified test protocol,” in Abstracts from the 64th Annual Scientific meeting of the Aviation, Space and Environmental Medicine Association (Toronto, ON) 23–27.

[B49] Moore S. T. Dilda V. MacDougall H. G. (2011). Galvanic vestibular stimulation as an analogue of spatial disorientation after spaceflight. Aviat. Space Environ. Med. 82, 535–542. 10.3357/asem.2942.2011 21614868

[B50] Muir S. W. Berg K. Chesworth B. Speechley M. (2008). Use of Berg Balance Scale for predicting multiple falls in community-dwelling elderly people: a prospective study. Phys. Ther. 88, 449–459. 10.2522/ptj.20070251 18218822

[B51] Mulavara A. P. Ruttley T. Cohen H. S. Peters B. T. Miller C. Brady R.. (2012). Vestibular-somatosensory convergence in head movement control during locomotion after long-duration spaceflight. J. Vestib. Res. 22, 153–166. 10.3233/VES-2011-0435 23000615

[B52] NeuroCom International, Inc (2007). Objective Quantification of Balance and Mobility. Clackamas, OR: NeuroCom International Inc.

[B53] Nicogossian A. E. Huntoon C. L. Pool S. L. Johnson P. C. (1988). Space Physiology and Medicine. (Vol. 19), 2nd Edn. Philadelphia, PA: Lea and Febiger.

[B54] Paloski W. H. Wood S. J. Feiveson A. H. Black F. O. Hwang E. Y. Reschke M. F. (2006). Destabilization of human balance control by static and dynamic head tilts. Gait Posture 23, 315–323. 10.1016/j.gaitpost.2005.04.009 15961313

[B55] Peterka R. J. Wall C. III Kentala E. (2006). Determining the effectiveness of a vibrotactile balance prosthesis. J. Vestib. Res. 16, 45–56. 16917168

[B56] Porte Y. Morel J. L. (2012). Learning on Jupiter, learning on the moon: the dark side of the G-force. Effects of gravity changes on neurovascular unit and modulation of learning and memory. Front. Behav. Neurosci. 6:64. 10.3389/fnbeh.2012.00064 23015785 PMC3449275

[B57] Rogers D. Van Syoc D. (2011). Clinical Practice Guideline for Motion Sickness. American Society of Aerospace Medicine Specialists. Virginia: The Aerospace Medical Association.

[B58] Roy J. E. Cullen K. E. (2001). Selective processing of vestibular reafference during self-generated head motion. J. Neurosci. 21, 2131–2142. 11245697 10.1523/JNEUROSCI.21-06-02131.2001 PMC6762599

[B59] Rupert A. H. (1998). “Haptics as the most intuitive spatial orientation system,” in Proceedings of the Third Annual Symposium and Exhibition on Situation Awareness in the Tactical Air Environment (Piney Point, MD), 3–7, 3–8.

[B60] Rupert A. H. (2000). Tactile situation awareness system: proprioceptive prostheses for sensory deficiencies. Aviat. Space Environ. Med. 71, A92–A99. 10993317

[B61] Rupert A. H. (2016). Post-Concussion Tools to Assist with Assessment, Treatment and Return to Duty. Final Project Report by Henry Jackson Foundation Report for Award W81XWH0920182. Fort Belvoir, VA: Defense Technical Information Center, (Houston, TX: NASA.

[B62] Rupert A. H. Guedry F. E. Reschke M. F. (1994). “The use of a tactile interface to convey position and motion perceptions,” in *AGARD Meeting on Virtual Interfaces: Research and Applications* (Houston, TX: NASA)

[B63] Rupert A. H. Lawson B. D. (2010). Initial consideration of the feasibility and optimal application of tactile sway cueing to improve balance among persons suffering from disequilibrium. USAARL Technical Report, Report No. 2011-01.

[B741] Rupert A. H. Lawson B. D. McGrath E. F. Wood S. J. (2011). Computerized posturography incorporating static and dynamic head tilts. *J. Vestib. Res.* 21, 73–75.

[B64] Shelhamer M. (2015). Trends in sensorimotor research and countermeasures for exploration-class spaceflights. Front. Syst. Neurosci. 9:115. 10.3389/fnsys.2015.00115 26321927 PMC4531325

[B65] Shepard N. Cole N. Bradshaw M. Hyder R. Parent R. McGrath B. J. (1998). Enhancing Sensitivity of the Sensory Organization Test (SOT) with the Head-shake (HS-SOT): Recommendations for Clinical Application. Clackamas, OR: NeuroCom International, Inc.

[B66] Taube J. S. Stackman R. W. Calton J. L. Oman C. M. (2004). Rat head direction cell responses in zero-gravity parabolic flight. J. Neurophysiol. 92, 2887–2997. 10.1152/jn.00887.2003 15212426

[B67] Tjernström F. Fransson P. A. Kahlon B. Karlberg M. Lindberg S. Siesjö P.. (2009). Vestibular PREHAB and gentamicin before schwannoma surgery may improve long-term postural function. J. Neurol. Neurosurg. Psychiatry 80, 1254–1260. 10.1136/jnnp.2008.170878 19574236

[B68] van Erp J. B. F. van Veen H. A. H. C. (2006). Touch down: the effect of artificial touch cues on orientation in microgravity. Neurosci. Lett. 404, 78–82. 10.1016/j.neulet.2006.05.060 16806701

[B70] Wall C. III. Kentala E. (2005). Control of sway using vibrotactile feedback of body tilt in patients with moderate and severe postural control deficits. J. Vestib. Res. 15, 313–325. 16614476

[B69] Wall C. III. Weinberg M. S. Schmidt P. B. Krebs D. E. (2001). Balance prosthesis based on micromechanical sensors using vibrotactile feedback of tilt. IEEE Trans. Biomed. Eng. 8, 1153–1161. 10.1109/10.951518 11585039

[B71] Welch R. B. Mohler B. J. (2014). “Adapting to virtual environments,” in Handbook of Virtual Environments: Design, Implementation and Applications, 2nd Edn., eds. K. S. Hale and K. M. Stanney, (New York, NY: CRC Press, an imprint of Taylor & Francis Group, LLC), 627–646.

[B72] Wood S. J. Loehr J. A. Guilliams M. E. (2011). Sensorimotor reconditioning during and after spaceflight. NeuroRehabilitation 29, 185–195. 10.3233/NRE-2011-0694 22027081

[B73] Wood C. D. Manno J. E. Manno B. R. Odenheimer R. C. Bairnsfather L. E. (1986). The effect of antimotion sickness drugs on habituation to motion. Aviat. Space Environ. Med. 57, 539–542. 3718377

[B74] Young L. R. Yajima K. Paloski W. (Eds) (2009). Artificial Gravity Research to Enable Human Space Exploration, (Cologne, Germany: International Academy of Astronautics), 1–37.

